# MicroRNAs as therapeutic targets in cardiovascular disease

**DOI:** 10.1172/JCI159179

**Published:** 2022-06-01

**Authors:** Bernhard Laggerbauer, Stefan Engelhardt

**Affiliations:** 1 Institute of Pharmacology and Toxicology, Technical University of Munich (TUM), Munich, Germany.; 2 DZHK (German Centre for Cardiovascular Research), Partner Site Munich Heart Alliance, Munich, Germany.

**Keywords:** Cardiology, Therapeutics, Cardiovascular disease, Noncoding RNAs

## Abstract

The discovery of microRNAs and their role in diseases was a breakthrough that inspired research into microRNAs as drug targets. Cardiovascular diseases are an area in which limitations of conventional pharmacotherapy are highly apparent and where microRNA-based drugs have appreciably progressed into preclinical and clinical testing. In this Review, we summarize the current state of microRNAs as therapeutic targets in the cardiovascular system. We report recent advances in the identification and characterization of microRNAs, their manipulation and clinical translation, and discuss challenges and perspectives toward clinical application.

Three decades ago, a small noncoding RNA in Caenorhabditis elegans was found to regulate gene expression at the posttranscriptional level (1, 2). What followed was the identification of numerous microRNAs (also termed miRNAs) in higher eukaryotes, and the finding that they regulate the majority of mammalian mRNAs (3). Nonetheless, the question how many microRNAs exist in humans is still a matter of debate. Of the 1973 human microRNAs annotated in mirBase 22.1 (4), many do not withstand curation for stringent criteria such as expression, sequence constraints, or evidence of productive precursor processing. Accordingly, the number of functional microRNAs in humans appears to range from 556 (mirGeneDB 2.0; ref. 5) to 758 (6). Since most microRNAs only show effects at sufficiently high expression in tissue (see below), this further reduces the fraction of functionally relevant microRNAs. A tentative assumption may thus be that up to 150 microRNAs have a critical role in the cardiovascular system. Of these, 30–35 microRNAs have been comprehensively analyzed and validated in experimental models in vivo (Table 1). The clinical development of many of these candidates has begun to reveal their potential, and several more candidates are expected to follow.

## Characteristics and features of microRNAs

### Biogenesis, stability, and strand bias of microRNAs

**Figure 1:**
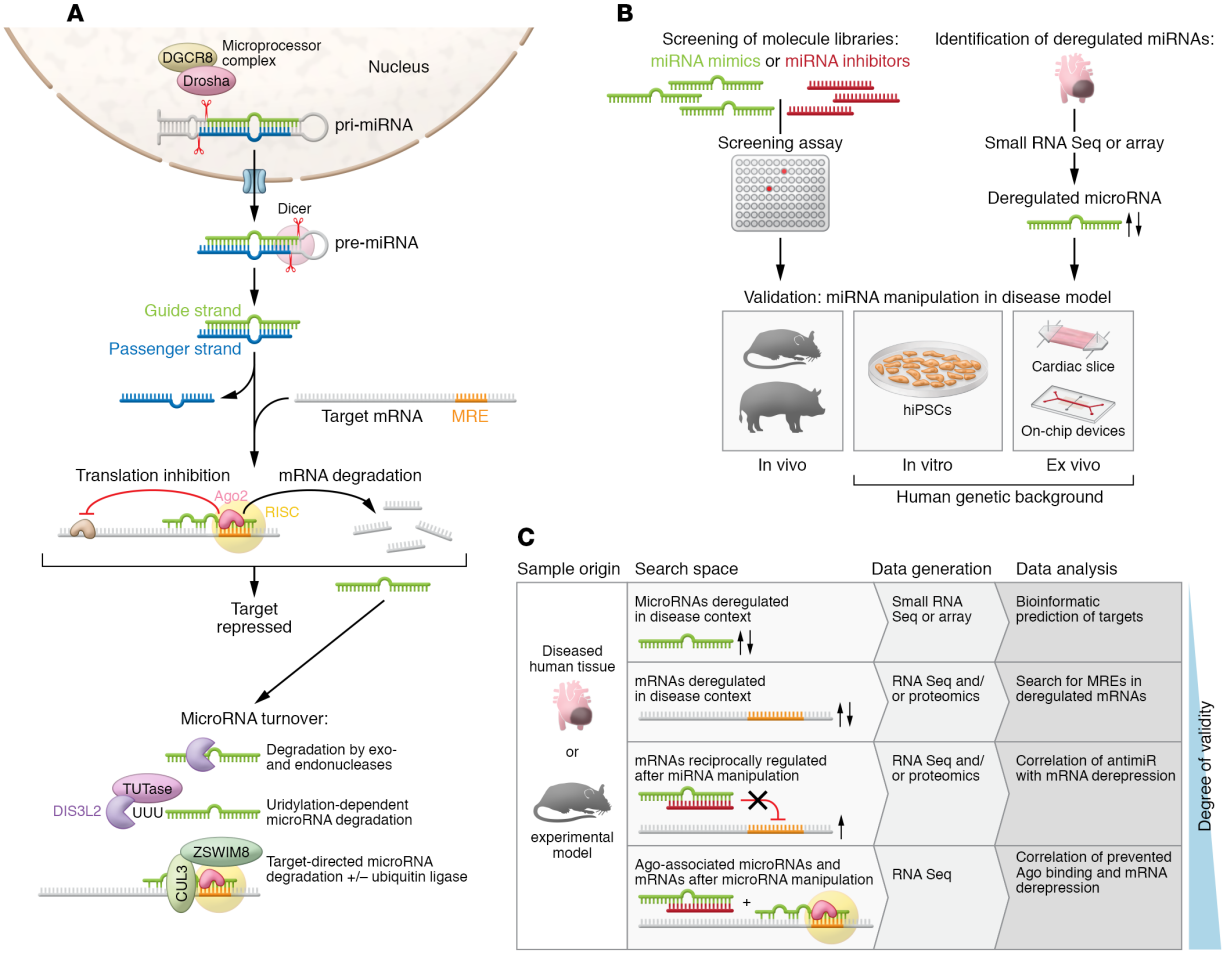
MicroRNA life cycle, and identification of microRNAs and their targets. (**A**) Canonical processing, functional activation, mechanism of action, and degradation pathways of microRNAs. Canonical microRNA biogenesis starts from larger hairpin RNA molecules (pri-miRNAs), which are generated by RNA Pol II transcription of microRNA genes or clusters, or which occur as part of introns. A microprocessor complex that contains the endonuclease Drosha, the DiGeorge critical region 8 protein (DGCR8), and other factors then cleaves these pri-miRNAs. The resulting pre-miRNA is exported to the cytoplasm, where the nuclease Dicer tailors it to 21 to 22 nucleotides in length. There are also noncanonical mechanisms of microRNA biogenesis, some of which bypass the microprocessor complex or Dicer. After processing to a duplex of 21–22 nucleotides in length each, one strand, termed the guide strand, becomes part of the RNA-induced silencing complex (RISC), whereas the passenger strand (or *-strand) undergoes accelerated degradation. If both strands are maintained, they can adopt individual functions, as demonstrated for cardiovascular miR-21 and miR-126 (11, 12). Another exception are microRNA strands that localize to the nucleus, where they function in unusual manners (12, 13). Degradation of microRNAs involves exonucleases XRN-1, PNPase old-35, and RRP41 (17) or the endonuclease Tudor-SN (154). The nuclease DIS3L2 degrades a subset of microRNAs after modification by terminal uridyltransferases (TUTases) (155). Mechanisms of target-directed microRNA degradation (TDMD) have been resolved, including the involvement of ubiquitin ligases (25, 26). (**B**) Routes toward the identification and validation of disease-relevant cardiovascular microRNAs. (**C**) Approaches for the identification of microRNA targets.

The biogenesis of microRNAs and their maturation have been addressed by excellent reviews (7, 8) and is illustrated in Figure 1A. After processing to a duplex of 21 to 22 nucleotides in length each, one strand, termed the guide strand, becomes part of the RNA-induced silencing complex (RISC), whereas the passenger strand (or *-strand) undergoes accelerated degradation (refs. 9, 10, and Figure 1A). If both strands are maintained, they can adopt individual functions, as demonstrated for cardiovascular miR-21 and miR-126 (11, 12). Another exception are microRNA strands that localize to the nucleus, where they function in unusual manners (12, 13).

Within the RISC, microRNAs associate with the endonuclease argonaute 2 (AGO2) and other proteins. Originally discovered in the context of RNA interference, the RISC can accommodate small interfering RNAs (siRNAs) or microRNAs, yet how they act therein differs. Whereas siRNAs require a full match to their target sequence, mammalian microRNAs depend only on a so-called seed sequence of 7 to 8 nucleotides that lies close to the 5′ end and has full target complementarity. Accessory pairing beyond the seed sequence can support target recognition, but only a fraction of microRNAs seem to depend on these interactions (8, 14, 15). MicroRNA target sites in mRNA (termed microRNA response elements, MREs) mostly lie within the 3′-UTR and less frequently in 5′-UTRs or coding regions (8, 16). Unlike lncRNAs or circRNAs, which can have various mechanisms of action, microRNAs have two clearly defined activities: they induce either degradation (the dominant activity) or translational silencing of target mRNAs (8).

The microRNA portfolio is complemented by nongenetic variants, termed isomiRs, which result from alternative microRNA processing, nucleotide addition, or editing (17, 18). Many cardiovascular isomiRs exist (19, 20), with levels fluctuating in disease (19). For the isomiRs of miR-487b-3p and miR-411-5p, distinct targetomes of variant and template have been revealed (21, 22).

At the other end of a microRNA’s life cycle stands its enzymatic degradation. Most microRNAs have considerable longer half-lives than mRNAs, yet there is considerable variability, depending on microRNA strand and sequence, cell type, and *trans*-acting factors (refs. 23, 24, and Figure 1A). Among the latter are also microRNA targets. Although mechanistic details of target-directed microRNA degradation (TDMD) have been resolved (8, 25, 26) and TDMD’s significance demonstrated in vivo (27), it is difficult to identify mRNAs that engage in TDMD.

## General approaches to manipulate microRNAs

### Synthetic oligonucleotides.

The unique mechanism of action of microRNAs makes them ideally druggable by synthetic oligonucleotides that mimic or inhibit their activity. MicroRNA mimics are applied as double strands to make them a substrate for Dicer and promote integration of one strand into the RISC. The use of single-stranded microRNA mimics (28) is more an exception than the rule. Antisense oligonucleotides (ASOs) for microRNA inhibitors (antimiRs) are applied as only a single strand and do not seem to become part of the RISC.

Chemical modification of oligonucleotides has become an imperative not only to reduce nuclease sensitivity and rapid renal clearance, but also to optimize delivery (29). This also applies to microRNA mimics and inhibitors (30, 31). Replacement of phosphodiesters by phosphorothioate is the most common backbone variation in therapeutic ASOs, prolonging serum half-life by orders of magnitude (31). Modification of the ribose 2′OH group, commonly by methyl (2′O-Me), methoxyethyl (2′O-MOE), or fluoro (2′-F), further increases stability (29, 31). The type, number, and positioning of modifications within microRNA mimics or siRNAs affects their ability to associate with the RISC. Whereas they mostly contain 2′O ribose modifications throughout the molecule, phosphorothioates are only tolerated at terminal positions (29, 32). AntimiRs, by contrast, typically contain multiple phosphorothioates, together with extensive ribose 2′O modification — either as MOE or F/MOE groups, or by alternating 2′deoxy ribose with 2′-to-4′ bridging nucleic acids that favor thermostable microRNA binding (31). Among these, locked nucleic acids (LNAs) are most frequently chosen because of their favorable nuclease stability, target affinity, pharmacokinetics, and tolerability (30). LNA antimiRs are composed of 12 to 16 nucleotides and contain interspersed DNA nucleotides in a number and positioning that promotes sequestration of microRNAs, but not RNase H cleavage. An extreme variation are tinymiRs composed of 8 seed-matching nucleotides, but their efficacy appears to fall behind that of longer antimiRs (33, 34). Cholesterol conjugation, a means to increase membrane penetrance, is part of the design of so-called antagomirs (35), also in the cardiovascular context (Table 2).

### Expression systems in vivo.

Next to synthetic molecules, expression systems offer additional opportunities when microRNA elevation or genetic inactivation is intended. Genetic methods to manipulate protein-coding genes, e.g., CRISPR/Cas9, transgenesis, or expression from plasmids, are also applicable to microRNAs. Adeno-associated viruses (AAVs) are particularly versatile tools for these purposes, and for therapeutic development; organotropic serotypes, long-lasting expression, and a favorable risk profile are strong arguments in their favor (36). Two AAV-based gene therapeutics have received market approval (voretigene-neparvovec, onasemnogene-abeparvovec), and others have demonstrated their efficacy in large-animal models, including cardiovascular disease models (37–39).

### Selecting microRNAs for therapeutic development.

Synthetic libraries of microRNA mimics or inhibitors can be the first step to select microRNAs by cell-culture-based screening (Figure 1B). Principally, these approaches either assess phenotypic effects or display which microRNAs can regulate a target of interest.

A fundamental advantage of functional screening is the possibility to identify microRNAs within their disease-relevant cellular context. Many phenotypic assays are relatively straightforward (e.g., assays for cell survival or morphology changes), quick, and adaptable to high throughput, as also demonstrated with cardiovascular cells (40–42).

Reporter assays, beyond their canonical use to validate MREs, are also suitable to identify microRNAs that regulate a defined mRNA. Typically, a cDNA for luciferase or a fluorescent protein is fused to the natural 3′-UTR of this mRNA, and exogenous microRNA mimic or inhibitor will then repress or relieve its expression. It should be cautioned that reporter assays usually ignore mRNAs with MREs that lie outside the 3′-UTR. Another limitation is that they do not mirror physiological microRNA-to-MRE stoichiometries and are thus at risk of errors.

Understanding microRNA deregulation in disease may further identify microRNAs with potential therapeutic relevance. Tissue samples from patients or from disease models in animals are valuable sources for these analyses (Figure 1B). Microarrays or small RNA sequencing (small RNA-Seq) are most widely used in this regard for the unbiased identification of microRNA candidates. Both enable a near-complete detection of basically any annotated miRnome (i.e., the entirety of microRNAs in a cell type or tissue).

## Functional validation in cardiovascular disease models

The cardiovascular system offers specific opportunities for the therapeutic development of microRNAs, such as the applicability of noninvasive methods (e.g., Doppler sonography or electrocardiography) and experimental models that faithfully recapitulate human cardiovascular disease.

Small animals, particularly mice, are still fundamental for the validation of microRNAs (Figure 1B) since their cardiovascular system shares substantial similarity with that of humans (43), and they are also a source of primary cells. However, some disparities between rodent and human cardiovascular systems (e.g., heart rate and contraction kinetics) or certain invasive routes of drug administration may necessitate large animal models (43). Pigs, for example, have been used in several microRNA-targeting cardiovascular studies (44–48).

Cells reprogrammed from human induced pluripotent cells (hiPSCs) contain the individual donor genome and are thus helpful for modeling human hereditary cardiovascular disorders. Cardiac myocytes obtained by reprogramming hiPSCs also form contraction-competent tissue suitable for drug testing (49). Tissue explants are another system with a human genetic background. Cardiac slices or aortic tissue from human patients maintain their organotypical features in culture (50, 51) and can be manipulated by viral transduction (52, 53), transfection (54), or used in coculture experiments (55, 56).

Omics technologies have become essential for microRNA characterization, since they examine, in an agnostic, unbiased manner, the effects of a microRNA throughout entire gene expression profiles and help to identify microRNA targets. RNA sequencing from lysed tissue (termed bulk RNA sequencing or RNA-Seq) combines this technology with relatively easy access to biosamples. One caveat of using tissue is that deregulated mRNAs might be obscured if the respective cell type is outnumbered by others where this mRNA is unaltered. In this case, magnetic cell separation (MACS), fluorescence-activated cell sorting (FACS), or a combination of both performed upstream of RNA-Seq enables the determination of cell-specific expression profiles and reveals low-abundance mRNAs.

Continuing this idea, single-cell RNA-Seq (scRNA-Seq) based on microfluidic separation and genetic barcoding offers the opportunity to determine transcriptomes of individual cells, e.g., from healthy (57) and failing human heart (58). For scRNA-Seq of miRnomes, a variety of work flows have been developed and validated in a comparative study (59), and we may expect that one or a few of them will become a broadly applied technical consensus.

Next to RNA-Seq, proteome analysis bears tremendous potential for the diagnosis and analysis of cardiovascular disease. Proteome data sets from patients have been generated from plasma and also from tissue (60–62). Proteome changes can now be assessed from single cells (63), allowing deep insight into pathophysiological changes. Some microRNAs suppress their targets at the level of translation, and proteomics would be able to identify these targets. The correlation of proteome and miRnome data can thus support and refine RNA-Seq data for a better understanding of microRNA-regulated networks.

### MicroRNA characterization by identification of mRNA targets.

For many years, bioinformatic prediction of seed-matching mRNAs has been the first step in microRNA target assessment (Figure 1C) (for completeness, it should be noted that noncanonical targets also exist; ref. 64). For most canonical microRNAs, tools like TargetScan predict multiple mRNA targets (3), which is expected, given the shortness of the seed region. An alternative to using the miRnome as a search space is to identify mRNAs that are deregulated in disease or upon microRNA manipulation (Figure 1C), followed by the analysis of MREs therein. A high degree of target validity can be established by coimmunoprecipitation of microRNAs with AGO-associated mRNAs, followed by sequence analysis. Comparing RNA-Seq data sets obtained with or without an antimiR can delineate mRNAs that have been derepressed from a microRNA as targets (65) (Figure 1C). For the validation of targets, their silencing or genetic inactivation, and in particular the mutation of their MREs, are important approaches.

What other parameters determine target recognition, aside from the mere presence of an MRE? With copy numbers in the range of 1 × 10^1^ to 1 × 10^5^ per cell (23, 66, 67), most microRNAs are in substoichiometric ratio to potential target sites in the transcriptome (67, 68). Only microRNAs with sufficiently high levels would thus be expected to cause measurable effects on targetomes (67–69). New perspectives came from evaluating the hypothesis that certain RNAs might function as competing endogenous RNAs (ceRNAs). Two models propose how a ceRNA could function as such; one postulates that an RNA must be present in excess or contain vicinal cooperativity-promoting MREs (67, 69). The other postulates that sequence context beyond the seed match creates high binding affinity (68), overruling unfavorable stoichiometry. Evidence of high-affinity sites was delivered by analyzing antimiRs for their ability to derepress targets (ref. 65 and Figure 1C). In support of this, dinucleotide motifs adjacent to MREs were found to contribute to the affinity for microRNAs (64). Despite their differences, the models seem to agree that (a) typically only highly abundant microRNAs confer far-reaching effects on targetomes, that (b) an individual target mRNA is usually unable to influence the expression of others, and (c) that additional MREs and/or sequence context contribute to target recognition.

Endogenous microRNA levels undergo extensive changes in disease, reaching from 3- to 4-fold up to 30-fold deregulation (70). This alone, or the simultaneous regulation of additional microRNAs, can have dramatic impact on targetomes and disease phenotype (71, 72). MicroRNAs that are cotranscribed as part of the same cluster can act in concert, as recently shown for the miR-106b~25 cluster (73). On the other hand, an individual microRNA may also regulate various levels of a cellular process. Examples of such multilevel regulation include miR-378a-3p, the miR-29 family, and miR-365-3p (54, 74, 75). In conclusion, activities in concert or on several layers add to the power of microRNAs as disease modifiers.

## Roles of microRNAs in the cardiovascular system

**Figure 2:**
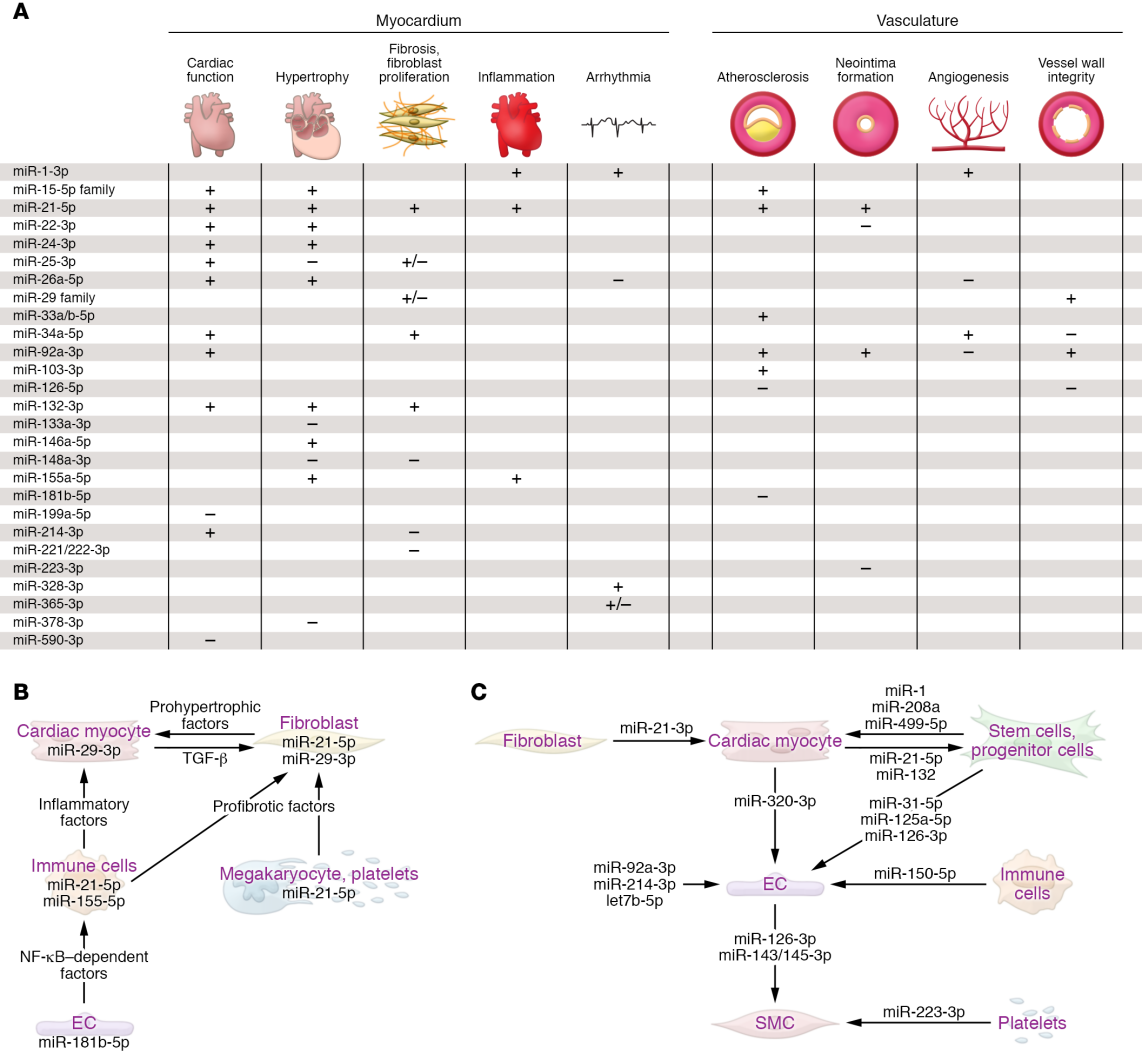
Functions of microRNAs in the cardiovascular system. (**A**) Table summarizing microRNA functions in myocardium and vasculature. + indicates that the process is promoted by the indicated microRNA, – indicates the pathophysiologic process is prevented by the indicated microRNA. Information on microRNAs that promote or impair cardiac function, after their elevation or inhibition, is provided in the respective column. (**B**) Exemplary microRNAs that control targets involved in cell-to-cell communication in the cardiovascular system (information compiled from studies cited in Table 1). (**C**) Paracrine roles of exemplary secreted microRNAs in the cardiovascular system. Atheroprotective effects are exerted by extracellular miR-126-3p (184) and miR-143-3p/miR-145-3p, proangiogenic effects are exerted by exosomal miR-143-3p, miR-222-5p, miR-92a-3p, and miR-214-3p, whereas miR-320-3p confers the opposite effect. The passenger (3′) strand of miR-21 is enriched in exosomes from cardiac fibroblasts, promoting cardiac myocyte hypertrophy (156), whereas the miR-21 guide strand, released by endometrial mesenchymal stem cells, is cardioprotective by promoting cell survival and angiogenesis (157). In the retrograde direction, several microRNAs of myocardial origin promote the mobilization of progenitor cells in bone marrow (102). miR-223-3p is delivered by platelets and regulates differentiation and proliferation of vascular SMCs (193). For an overview on these and other cardiovascular microRNAs with proposed paracrine function, see refs. 99–101. EC, endothelial cell; SMC, smooth muscle cell.

Although GWAS identified polymorphisms in microRNA biogenesis factors, microRNA genes, or MREs (76), pathophysiological consequences have been resolved for few. Against this limited knowledge stands a wealth of microRNAs that are deregulated or modified in disease. But does the deregulation of a microRNA cause disease or merely indicate it? MicroRNAs that play active roles in pathophysiology frequently combine high basal expression at steady state, pronounced deregulation in disease (Table 1), and preferential occurrence of both in cells/tissue. For example, miR-21-5p is the most abundant microRNA in cardiac macrophages and is 7-fold upregulated in myocardium of the transverse aortic constriction (TAC) model of ventricular pressure overload (77), and miR-29b-3p is highly expressed in cardiac myocytes and approximately 3-fold upregulated upon TAC (75). Many of the 30 to 35 microRNAs with strong in vivo evidence of critical cardiovascular roles (Table 1) cause distinct pathophysiological effects in myocardium or vasculature when manipulated (Figure 2A). A fraction of these do so by engaging signaling pathways that lead to the secretion of protein factors (Figure 2B), whereas others are themselves part of extracellular vesicles, in particular exosomes (Figure 2C). Parallel to this growth of knowledge, the therapeutic development of microRNAs in myocardium and vasculature has markedly increased (ref. 78 and Tables 1 and 2). Although space limitations restrict us from detailed discussion of all microRNAs in Table 1, we highlight some candidates with regard to their cardiovascular roles and clinical development:

miR-21-5p is strongly upregulated in the failing human heart (79) and also in diseases of kidney and lung that share fibrosis as their common denominator. miR-21 inhibitors prevent cardiac fibrosis (79) or neointima formation (80) in animal models. Whereas a global miR-21-5p deficiency remained silent (33, 81), the effects of inhibitors were recapitulated by a genetic miR-21 knockout in nonmyocyte cells (81), indicating a crucial role therein. Cardiac fibroblasts and macrophages display the highest miR-21-5p levels (77, 81). Mice with macrophage-specific miR-21-5p deficiency were resistant to TAC-induced structural and functional phenotypes, along with reduced inflammation (77). Consistently, pigs that received antimiR-21 after ischemia/reperfusion had better cardiac function and reduced inflammation (44). Together, this suggests a strong profibrotic and proinflammatory function of miR-21-5p in myocardium. In agreement, LNA-antimiR-21 is currently also being tested in a phase II study for the treatment of a fibrotic kidney disease (Table 2).

miR-29 is a family of four almost identical variants. Its ability to regulate collagens and other matrix proteins makes it a designated target for antifibrotic therapies. One of the first studies on miR-29 in this regard demonstrated collagen repression and improved cardiac function by miR-29 mimics (82). Since then, this concept was recapitulated in other organs, culminating in the development of a miR-29 mimic (MRG-201) to treat idiopathic pulmonary fibrosis (Table 2). Following this principle, but with the aim to derepress collagen expression, antimiR-29b supported vascular wall stabilization in mouse models of abdominal aortic aneurysms (83, 84). Distinct from these findings, our observation that inhibition, rather than elevation, of miR-29 prevents cardiac fibrosis (75) must have surprised the community, but its underlying mechanism has been resolved; each of the miR-29 variants is predominantly expressed in cardiac myocytes (high levels in cardiac fibroblasts occur only with prolonged cultivation), within which miR-29 executes its primary role by engaging the Wnt pathway for cellular hypertrophy and paracrine, profibrotic signaling to fibroblasts. Thus, other than skin diseases where miR-29 elevation is beneficial to suppress fibrotic pathways in fibroblasts, the inhibition of miR-29 appears appropriate in myocardium.

miR-92a-3p is highly expressed in endothelial cells and deregulated in mouse models of vascular and myocardial tissue injury (45, 85). An LNA antimiR against miR-92a promoted angiogenesis and tissue repair in these models (85), which was later confirmed in a pig model of ischemia/reperfusion (45). First steps in clinical translation have been made with a pharmacological study on antimiR-92a (termed MRG-110) in healthy individuals who had received a single i.v. injection (86). Of note, since intradermal injection of antimiR-92a was also effective in animal models of skin injury, a second phase I clinical study was conducted for this route of administration (ClinicalTrials.gov NCT03603431).

miR-132-3p has moved from preclinical to clinical translation at impressive speed. Genetic deficiency of the miR-132/-212 cluster or an antagomir against miR-132-3p prevented TAC-induced pathologic cardiac remodeling (87). Based on this, inhibition of miR-132-3p has been developed in mouse models of heart failure and chronic pressure overload (46, 87). A study in a pig model of heart failure demonstrated long persistence in cardiac tissue (*t_½_* of 3 weeks) and an advantageous safety profile, and validated the derepression of miR-132 targets (46). Also in pig models, antimiR-132 improved cardiac function after myocardial infarction (MI) (88) or under chronic pressure overload (89). A first-in-human, dose-escalating study (phase Ib) in heart failure patients revealed good tolerability and first evidence of a therapeutic benefit (90).

miR-155-5p expression in immune cells is upregulated in patients with cardiac inflammation or respective animal models (70, 91). Bone marrow transplantation experiments in mice attributed the proinflammatory activity of miR-155 to macrophages (91), where it enhances NF-κB expression and, in that, opposes miR-146a-3p (92). miR-155 inhibition ameliorates cardiac inflammation in mice (70, 91), albeit the finding that macrophage-specific miR-155 deficiency obstructs arteriogenesis after vascular injury (93) demands further analyses in this regard. An antimiR against miR-155 (cobomarsen) has passed a phase I study on cutaneous T cell lymphoma (ClinicalTrials.gov NCT02580552) (94), but a phase II study was terminated for strategic reasons. The gathered clinical data would be valuable to guide the development of antimiR-155 for cardiovascular therapy.

### Circulating microRNAs as mediators of cellular communication

Many cardiovascular disease conditions alter the serum levels of microRNAs, a phenomenon that spurs their utilization as biomarkers (for reviews on these developments, see refs. 95–97).

Intriguingly, microRNAs are also actively released by cells, mostly in the form of exosomes. Whereas vesicular microRNAs may likewise serve as biomarkers, they also hold therapeutic potential. The broadest evidence for therapeutic effects of vesicular microRNAs has been established in the context of cancer, stem cells and progenitor cells of various origins, or cardiovascular disease (98). Multiple microRNAs are exosomal cargo in cardiovascular disease, and for many, the donor and recipient cells, and the effects elicited therein, have been described (refs. 99–101 and Figure 2C). Interestingly, exosomes appear to be enriched for microRNA passenger strands that, in the case of miR-21-3p, can adopt paracrine function (11). As part of exosomes, cardiac microRNAs can affect remote tissues, for example bone marrow (102). However, the relatively small amounts of microRNAs in plasma also raise concerns about their signaling power (103), prompting more evidence from studies with deficiency of individual microRNAs. Also, the preparation of native or engineered exosomes with defined microRNA content remains challenging (104), as is the improvement of tissue delivery (105).

## MicroRNA-targeting therapy on its way to the clinic

### The scope of oligonucleotide-based therapies.

Most oligonucleotides in therapeutic development are designed to inhibit targets through reverse complementary (antisense) base pairing. This includes ASOs that induce cleavage by RNase H, morpholinos that mask translation initiation or splice sites, siRNAs, and microRNA inhibitors (29). Currently, 10 siRNA- or other ASO-based drugs are approved, with several more in clinical studies. Inclisiran, an siRNA that reduces LDL cholesterol and prevents atherosclerosis, may be viewed as the first-in-class ASO for the treatment of cardiovascular disease.

AntimiRs against miR-122-5p (miravirsen, RG-101) for the treatment of hepatitis C have long been the most advanced microRNA-based drug candidates (Table 2). Although their medical need has faded due to the outstanding efficacy of other drugs and the gradual development of viral resistance (106), these antimiRs showed that microRNA-based therapy in patients is possible. By the beginning of 2022, 19 clinical studies on microRNA-based therapeutics had been completed or were ongoing (Table 2). Additional studies, two on miR-103/107-3p (AZD4076) and one on miR-155-5p (cobomarsen) were terminated or halted by the sponsor for strategic reasons.

### Clinical status of microRNA-based cardiovascular therapy development.

Despite the abandoning of several microRNA-targeting therapeutic developments in other indications (e.g., miravirsen, RG-101, cobomarsen, AZD4076), its impact on the cardiovascular field seems smaller than presumed. Preclinical and clinical data obtained with inhibitory oligonucleotides — even those that have been discontinued — provide valuable information for the design and performance of microRNA-targeting cardiovascular therapies. This applies to miR-17-5p, miR-21-5p, miR-29b-3p, and miR-92a-3p, which have been extensively studied in the laboratory and the clinic (Table 2), but particularly to the aforementioned miR-132-3p inhibitor (CDR132L), developed to treat heart failure. Currently planned for phase II testing, CDR132L might become the first microRNA-targeting drug in cardiovascular therapy.

Other than ASOs, no application of microRNA mimics or overexpression for cardiovascular indications seems close to clinical application. Given the aforementioned complications observed in the antitumoral study on MRX-34 (107) or the adverse effects of prolonged miR-199a expression in mouse models (47), timing and dosing seem particularly critical in microRNA-elevating therapy.

## Open questions and major challenges

### Cardiovascular delivery and tropism of oligonucleotides

**Figure 3:**
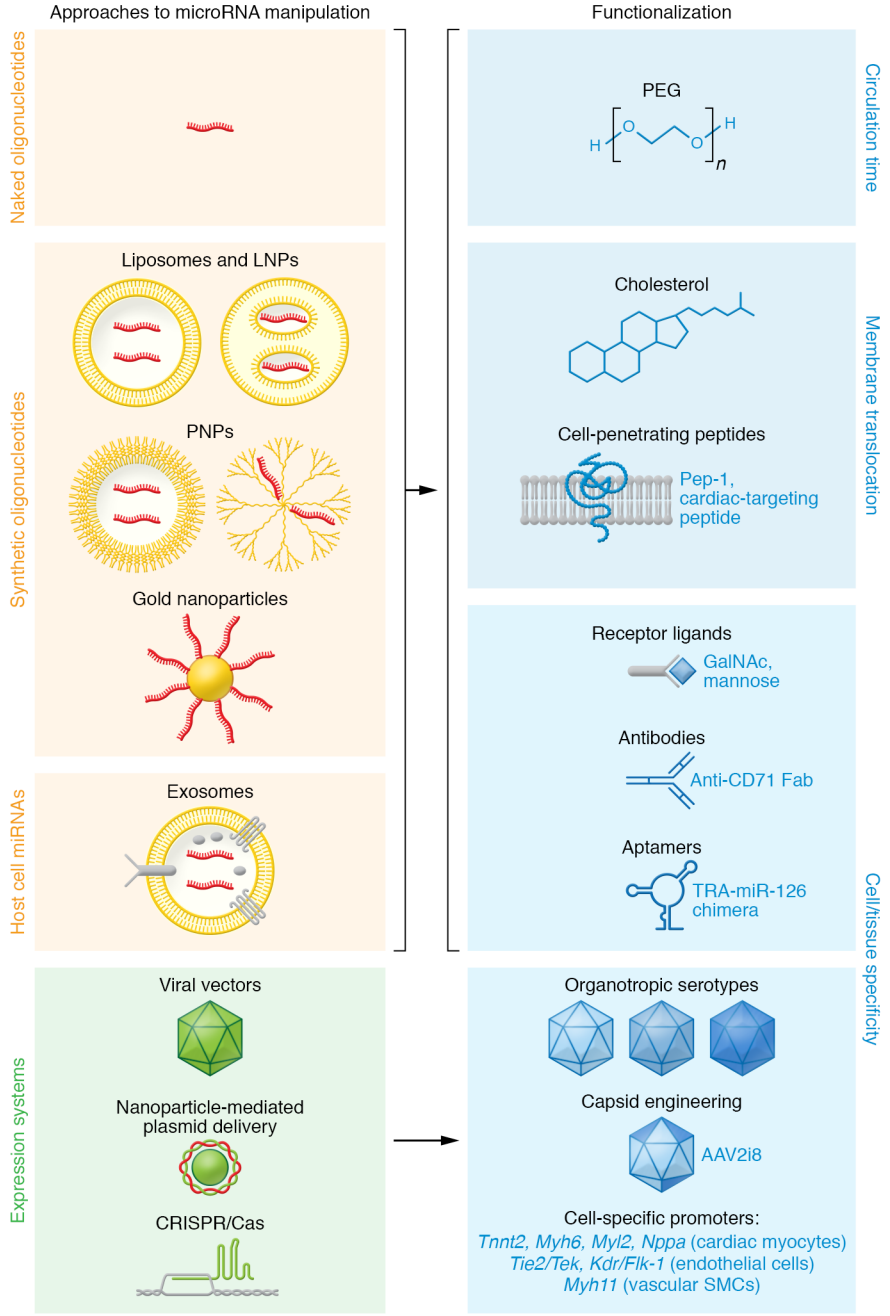
Molecular vehicles for microRNA modulators and their functionalization. Improved nuclease resistance by the use of modified nucleotides in synthetic oligonucleotides allows for application as “naked” molecules (112). Their embedding in liposomes or lipid nanoparticles (LNPs) or polymer-based nanoparticles (PNPs) can improve cell entry via endocytosis (113). Metal particles such as gold have been used as carriers for oligonucleotides (113) and plasmids (126). Exosomes with microRNA cargo can be isolated from native sources or engineered for optimized microRNA loading or cell specificity (104, 105). Oligonucleotides or their carriers can be further functionalized by conjugation to improve their circulation time (e.g., by PEGylation), membrane penetrance (e.g., cholesterol, cell-penetrating peptides), or to enhance their cell- or tissue-specific delivery (e.g., by coupling to receptor ligands, antibody fragments, or aptamers). TRA, transferrin receptor aptamer. Viral vectors and their organotropic serotypes, particularly adeno-associated virus (AAV), can be utilized for the expression or genetic inactivation (e.g., using CRISPR/Cas systems) of microRNAs or their targets. Improved transduction and/or tropism can be achieved by engineering AAVs (122, 123), and the use of cell-type-specific promoters adds further improvement. Exemplary promoters are denoted for gene expression in cardiac myocytes (*Tnnt2*, cardiac troponin T2; *Myh6*, myosin heavy chain 6; *Myl2*, myosin light chain 2; *Nppa*, natriuretic peptide A), in endothelial cells (protein tyrosine kinase *Tie2*/*Tek*; *Kdr*/*Flk-1*, kinase insert domain receptor/fetal liver kinase 1), and in vascular smooth muscle cells (*Myh11*, myosin heavy chain 11). For a critical review on endothelial cell–specific promoters, see Chakraborty et al. (158).

The establishment of effective oligonucleotide concentrations in target tissue or cells is a challenge that inspired a panel of strategies (summarized in Figure 3), of which many hold promise for cardiovascular applications. Due to their hydrophilic nature, oligonucleotides do not penetrate membranes well. Their distribution into cardiovascular tissue is potentially outcompeted by renal filtration (108). Moreover, the fenestration of the endothelium in liver and high monocyte numbers in spleen and bone marrow reduce the cardiovascular availability of oligonucleotides (109). In myocardium, this leads to comparably small cellular uptake (110), although disease conditions apparently improve this process (111). A further, more general problem oligonucleotides face is their spatial seclusion in endosomes after endocytosis, which they must escape to reach their targets (109). LNA antimiRs partly circumvent these obstacles, since they penetrate membranes as “naked” molecules (112), and indeed, many cardiovascular studies go without formulation of the antimiR (see Table 3).

Nonetheless, a large variety of formulation or conjugation strategies has been developed to increase circulation time, membrane translocation, intracellular availability, or tissue tropism of oligonucleotides (29). Nanoparticles based on lipids, polymers, a combination of both, or metals serve as carriers of oligonucleotides (ref. 113 and Figure 3). Conjugation to polyethylene glycol (PEG), a common strategy to slow drug elimination, is also applied for oligonucleotides.

Cholesterol can not only be conjugated to oligonucleotides to facilitate their membrane translocation, but also to nanoparticles. Cell-penetrating peptides (CPPs), including a cardiac-targeting peptide, have proven their suitability in cardiovascular disease models in vivo (114, 115). Currently, a CPP conjugate of eteplirsen is being investigated in a phase II clinical study for the treatment of Duchenne muscular dystrophy (ClinicalTrials.gov NCT04004065).

The highest cellular tropism would be expected from coupling oligonucleotides or microRNA vehicles to receptor ligands or other cell-targeting molecules (ref. 116 and Figure 3). Molecules that bind to cell surface proteins qualify as coupling partners of oligonucleotides, provided that they do not hamper translocation or activity of the drug or cause side effects. One development along this strategy is an siRNA coupled to a CD71 Fab′ fragment that targets heart and skeletal muscle in mice and was therapeutically effective in muscular dystrophy (117). Potential also lies in centyrins — derivatives of fibronectin 3 that can be engineered for specificity and affinity, and be coupled to oligonucleotides (118). A folate-coupled antimiR against miR-34-3p preferentially targets tumors in mice (119). More clinically advanced are oligonucleotides conjugated to N-acetylgalactosamine (GalNAc), a natural ligand of the asialoglycoprotein receptor 1, which is strongly expressed on hepatocytes and thus ideal for liver-targeted therapies (Table 2). We may expect various other sugars to prove suitable for cell-specific oligonucleotide delivery, such as mannose, the receptor for which is predominantly found on macrophages. Finally, several aptamers have been tested in combination with siRNAs (120), and one promoted miR-126-3p delivery through binding to the transferrin receptor (121).

Among viral vectors as vehicles for genetic information, AAVs stand out for their panel of organotropic serotypes that can be further optimized, for example by capsid engineering (122, 123). An example is the chimera of an AAV2 inner loop mutant and AAV8 (AAV2i8, alias BNP116) that preferentially transduces myocytes (124). This vector was utilized to express constitutively active inhibitor-1 in a pig model of cardiac ischemia (38) and is currently being tested in a phase I clinical study (ClinicalTrials.gov NCT04179643). Recently, directed evolution yielded AAVs with superior specificity for muscle cells and high transduction efficiency (125). Promoters for specific gene expression in various cardiovascular cell types (Figure 3) further expand the possibilities.

Apart from the advantages viral vectors offer, some molecular genetic tools such as CRISPR/Cas plasmids can also be delivered without the help of viruses (Figure 3), e.g., by transfection. Whether the nanoparticle-based delivery of plasmids for noncoding RNA (Figure 3), as shown for a circRNA construct (126), will prove suitable for microRNA expression remains to be tested.

#### Routes of administration.

With tissue-specific oligonucleotide modifications yet to progress into late-stage therapy development, the route of administration retains high importance for improved efficacy. Intravenous administration of oligonucleotides is most widely applied in experimental models, and also in the phase Ib study on antimiR-132 (Table 3). It should be cautioned, however, that i.v. injection rapidly dilutes the drug, and the aforementioned fenestration of certain noncardiovascular tissues adds to this problem. Intraperitoneal injection has been applied in cardiovascular preclinical studies (46, 127, 128), and intracardial injection has been applied in rodents (28, 129), yet the risks of either administration disfavor application in humans. Subcutaneous or intradermal application of oligonucleotides has been successful in cardiovascular studies in mice (111, 130, 131) and monkeys (132). Their minimally invasive character and advantageous pharmacokinetics (110) make them favorable for microRNA-based drugs (Table 3). It should be noted, however, that skin reactions at the injection site frequently occurred in clinical studies (133) (see below for immunogenicity). Several studies have employed device-based methods to combine the advantage of local drug delivery and a low risk of tissue injury. Coronary catheterization, today clinically routine, has been used for the delivery of microRNA drugs in small (79) and large animals (44, 45, 111).

#### Dosing regimens.

Most microRNA mimics or inhibitors tested in cardiovascular disease models in vivo are applied in consecutive doses within hours to days after disease induction (Table 3). Where tested, therapeutic effects by LNA antimiRs appeared within 2 or 3 days (131, 134, 135). Endowed with improved nuclease stability, microRNA modulators display typical half-lives of 3 weeks in cardiac tissue, allowing for effect durations of at least 18 to 46 days in mice (28, 75, 79) or 28 days in pigs (46) (the end points of these studies). An impressive effect duration of approximately 4.5 months was observed with antimiR-loaded nanoparticles, yet it is unclear whether this is attributable to the formulation format (136). The siRNA drug inclisiran provides therapeutic efficacy with only one or two subcutaneous injections per year. This exciting finding should encourage the development and testing of microRNA drugs with similar properties and pharmacokinetics. Another aspect is that microRNA mimics or inhibitors not only have the potential to treat, but also to prevent, cardiovascular disease. For example, mice that received LNA-antimiR-26a prior to MI showed milder phenotypes and better revascularization (131).

### Evaluating the risk of side effects

#### Immune reactions.

There are three potentially immunogenic factors to be considered in RNA-based therapy: (a) the nucleotide moiety or its chemical modification, (b) drug formulants, and (c) vectors used for overexpression. A phase I study using a miR-34 mimic against refractory cancer was abandoned due to fatal immune reactions (107), but it is unclear which of the drug components caused this. Likewise, immune responses seen with certain ASOs (137) are not fully resolved. These occurrences are contrasted by very promising safety data from many other clinical studies (Table 2).

Our innate immune response recognizes oligonucleotides as pathogen-associated molecular patterns (PAMPs). Toll-like receptors (TLRs) are a family of pattern recognition receptors (PRRs) that sense double- and single-stranded oligonucleotides. However, replacing individual nucleotides can reduce the immunogenicity of an siRNA without loss of efficiency (138). Analogously, naturally occurring nucleoside modifications help to evade TLR recognition (139), as well as 2′O-Me (140) or LNA modifications (141). The immunotolerance for LNA antimiRs is thus best explained by the presence of this moiety.

Formulation in nanoparticles can shield oligonucleotides from PRRs, and PEG in oligonucleotide drugs is used for this purpose in addition to the benefit of increased circulation time. PEG, however, induces antibodies that in one case have been made accountable for severe adverse effects (142). The possibility of PEG-related safety issues must be taken seriously, despite a long list of well-tolerated, approved PEGylated drugs.

Since viral vectors are potentially immunogenic, there is in principle a chance for adverse effects and for the evocation of neutralizing antibodies (if not present a priori). These hurdles gave rise to engineered “stealth” viruses with reduced immunogenicity (123). Moreover, approved virus-based gene therapeutics are typically combined with immunosuppressants.

#### Toxicity.

Oligonucleotide drugs could, in principle, confer toxicity by sequence-dependent or sequence-independent mechanisms, in this case caused by chemical modifications. The latter has been primarily observed with certain gapmers, apparently due to their strong protein binding (143). MicroRNA mimics or antimiRs differ from gapmers by a more uniform distribution of modifications at the 2′-ribose position. This may explain in part why the majority of preclinical and clinical studies on microRNA mimics or antimiRs reported good safety and tolerance (see refs. 90, 106, 144 for examples).

Sequence-dependent toxicity of antimiRs has been observed in high doses (>80 mg/kg), independent of their chemical modification (127) (note that antimiRs in clinical development are applied at lower dose and with favorable risk profiles; see Table 3). A plausible mechanism is that antimiRs, by preventing their respective microRNAs from AGO binding, allow other microRNAs to take their position in the RISC (145). Analogously, microRNA mimics, in large excess, can outcompete endogenous microRNAs from entering the RISC (146) or bind nonspecifically to RNAs. Whether this accounts for the unexplained complications seen in the miR-34–mimic study (107) remains to be clarified.

#### Tumorigenesis.

Many microRNAs involved in cardiovascular diseases have also been proposed to function in cancer (147). It has become increasingly clear that heart failure and cancer share pathophysiologic mechanisms (148), raising the question whether interfering with specific microRNAs may be beneficial for the treatment of both diseases. Some evidence in support of this hypothesis has been elaborated; beyond their therapeutic cardiovascular effects, antimiRs against miR-21-5p, miR-146a-5p, or miR-155-5p also prevented tumor growth in the respective mouse models (149–151). Others, such as miR-92a-3p, seem to be far less critical in cancer than members of their genetic cluster (152). However, since continuous, uncontrolled cardiac overexpression of miR-199a in pigs induced the formation of weakly differentiated myoblasts, causing fatal arrhythmia (47), this must be considered in risk assessment. Several microRNAs with well-documented cardiovascular function have been assigned an oncogenic or tumor suppressive role solely based on cell culture assays, expression data, or target predictions. Thus, long-term evaluation in animal models and analyses of tissue beyond the cardiovascular system should help to assess the risk of tumorigenesis.

## Future perspectives

The growing number of clinical studies targeting microRNAs, leading up to the first clinical study of an antimiR in cardiovascular therapy, is a clear testimony of the progress made in the past decade. The fact that many microRNAs are yet to be characterized leads us to presume a wider scope of disease conditions and applications of microRNA therapeutics than is currently visible. As recently laid out in a critical evaluation of the vast number of descriptive publications on microRNAs (153), the field is called upon to validate the function of microRNA candidates with scrutiny. Combining microRNA manipulation in disease models, omics technologies, and thorough preclinical testing will be key to improve the therapeutic development and reduce the risk of dropouts.

Although the development of synthetic oligonucleotides has mastered major hurdles, the delivery of these molecules still poses considerable challenges. This holds true in particular for cardiovascular tissue, which does not take up oligonucleotides efficiently. Ideally, certain application routes, e.g., local catheter-based delivery, will become dispensable once the pharmacokinetics of oligonucleotides are further improved. Another hope is that oligonucleotides will be modified not only for improved uptake, but also for cell specificity. This largely underdeveloped area will require intense efforts for the screening of ligands and their chemical coupling to oligonucleotides, together with methods that assess cellular oligonucleotide concentrations.

## Figures and Tables

**Table 3:**
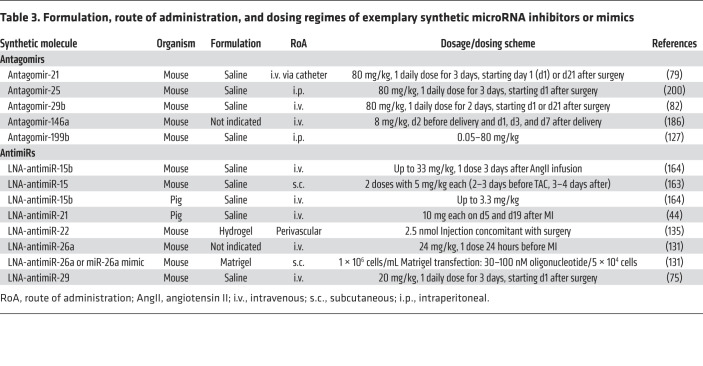
Formulation, route of administration, and dosing regimes of exemplary synthetic microRNA inhibitors or mimics

**Table 2:**
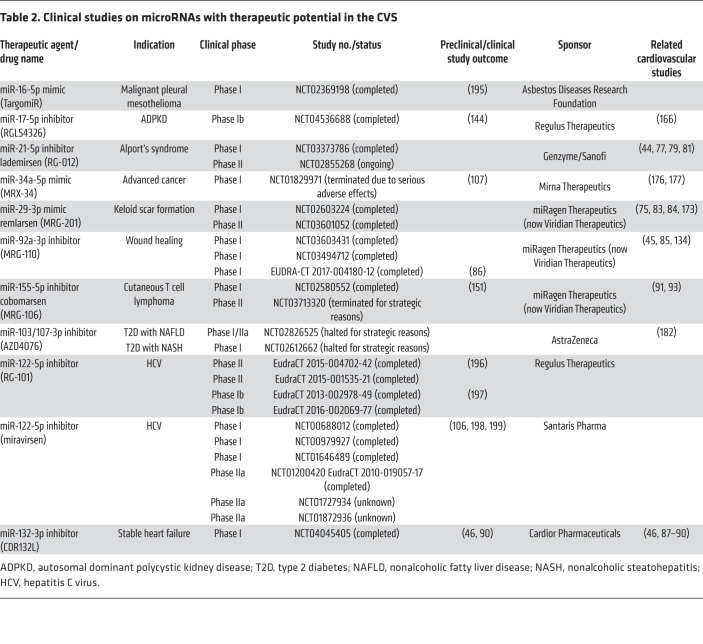
Clinical studies on microRNAs with therapeutic potential in the CVS

**Table 1:**
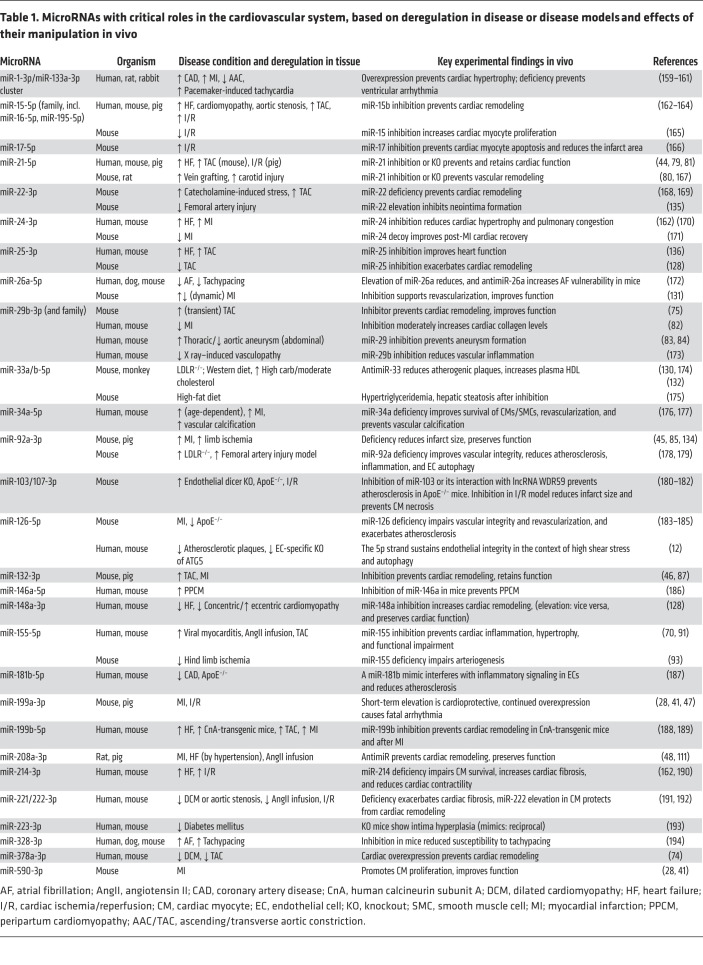
MicroRNAs with critical roles in the cardiovascular system, based on deregulation in disease or disease models and effects of their manipulation in vivo

## Version 1 06/01/2022

Electronic publication
